# Vascular endothelial growth factor B (VEGF-B) is up-regulated and exogenous VEGF-B is neuroprotective in a culture model of Parkinson's disease

**DOI:** 10.1186/1750-1326-4-49

**Published:** 2009-12-10

**Authors:** Torsten Falk, Shiling Zhang, Scott J Sherman

**Affiliations:** 1 College of Medicine, University of Arizona, Department of Neurology, Tucson, AZ 85724, USA

## Abstract

Parkinson's disease (PD) results from the degeneration of dopaminergic neurons in the substantia nigra and the consequent deficit of dopamine released in the striatum. Current oral dopamine replacement or surgical therapies do not address the underlying issue of neurodegeneration, they neither slow nor halt disease. Neurotrophic factors have shown preclinical promise, but the choice of an appropriate growth factor as well as the delivery has proven difficult. In this study, we used a rotenone rat midbrain culture model to identify genes that are changed after addition of the neurotoxin. (1) We challenged rat midbrain cultures with rotenone (20 nM), a pesticide that has been shown to be toxic for dopaminergic neurons and that has been a well-characterized model of PD. A gene chip array analysis demonstrated that several genes were up-regulated after the rotenone treatment. Interestingly transcriptional activation of vascular endothelial growth factor B (VEGF-B) was evident, while vascular endothelial growth factor A (VEGF-A) levels remained unaltered. The results from the gene chip array experiment were verified with real time PCR and semi-quantitative western analysis using β-actin as the internal standard. (2) We have also found evidence that exogenously applied VEGF-B performed as a neuroprotective agent facilitating neuron survival in an even more severe rotenone culture model of PD (40 nM rotenone). VEGF-B has very recently been added to the list of trophic factors that reduce effects of neurodegeneration, as was shown in an *in vivo *model of motor neuron degeneration, while lacking potential adverse angiogenic activity. The data of an *in vivo *protective effect on motor neurons taken together with the presented results demonstrate that VEGF-B is a new candidate trophic factor distinct from the GDNF family of trophic factors. VEGF-B is activated by neurodegenerative challenges to the midbrain, and exogenous application of VEGF-B has a neuroprotective effect in a culture model of PD. Strengthening this natural protective response by either adding exogenous VEGF-B or up-regulating the endogenous VEGF-B levels may have the potential to be a disease modifying therapy for PD. We conclude that the growth factor VEGF-B can improve neuronal survival in a culture model of PD.

## Findings

The two most pressing therapeutic challenges in PD are to (1) provide a stable level of dopamine replacement and (2) slow disease progression [1-4]. Neurotrophic growth factors such as the glial-derived neurotrophic factor (GDNF), neurturin, FGF-2 and others, have shown great promise in experimental models of PD [5,6]. The hope is that using these factors in human PD could provide a potent disease-modifying therapy; however, clinical development of these agents is problematic [5]. Intracerebroventricular administration of GDNF via a micro pump [7] and neurturin delivery via viral vector-mediated gene transfer [8] ultimately failed in Phase II clinical trials. These disappointing results despite robust preclinical data could be due to problems with the delivery method or choice of neurotrophic factor.

One path to identify new potential modifiers of PD is by using gene chip arrays utilizing *in vitro *and *in vivo *models of PD. In this study, to identify candidate genes, we challenged rat midbrain cultures with rotenone, a pesticide that has been shown to be toxic for dopaminergic neurons and that has been a well-characterized model of PD [9,10].

Timed-pregnant Sprague-Dawley rats were anesthetized by exposure to CO_2 _and sacrificed. Fetuses were removed at E17, anesthetized by cooling on ice, decapitated, and the midbrain was dissected. Details of the methods have been reported [11,12]. Tissue culture media and sera were obtained from Gibco-BRL, Grand Island, NY. The procedure was approved by the IACUC at the University of Arizona and conformed to the guidelines of the National Institutes of Health. The number of animals used and their suffering was minimized. We developed protocols *in vitro *using rotenone (Sigma-Aldrich, St. Louis, MO) to produce damage to dopaminergic neurons by adding it at the indicated concentrations at day 6 in culture. In previous work [11], an initial rotenone concentration response curve was established and the LD50 for 5 day exposure was found to be 25-50 nM. We chose to look at a slightly lower concentration of rotenone (20 nM), since we were interested in changes in mRNA before the cells are lost. We isolated the mRNA of 11 day old cultures 5 days after the rotenone challenge, and of untreated control cultures, before performing a gene array analysis (n = 3 separate experiments). The RNA isolation was done with the Qiagen RNA kit (Qiagen, Valencia, CA), using the manufacturers protocol. The gene array analysis was carried out using the GeneChip Rat Genome 230 2.0 Array (Affymetrics, Santa Clara, CA) and standard procedure. Data was analysed using the limma software package [13,14].

**Figure 1:**
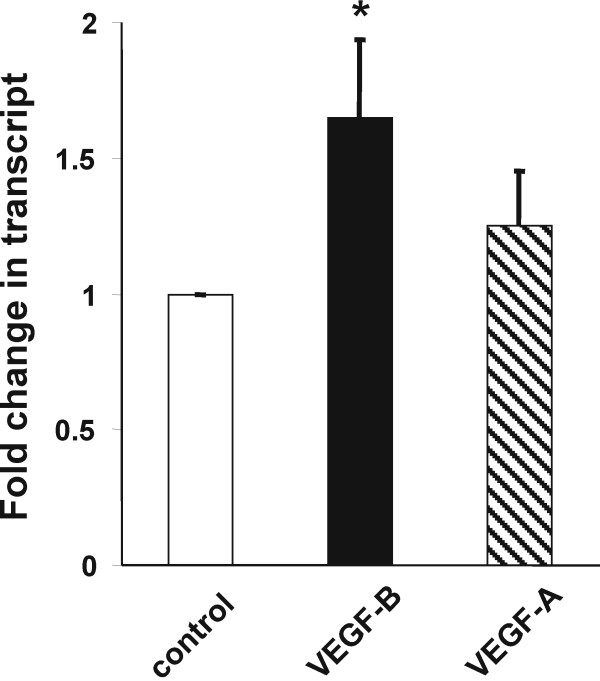
**Up-regulation of VEGF-B transcript, but not of VEGF-A transcript, after rotenone (20 nM) treatment of midbrain cultures**. The fold-increase of transcript vs. control as determined with real time PCR is shown as mean ± s.e.m for VEGF-B (black bar) and VEGF-A (shaded bar); n = 7 separate experiments. Mean fold changes in rotenone-treated cultures compared to control (± s.e.m.) were: VEGF-B = 1.65 ± 0.29; P = 0.026; VEGF-A = 1.25 ± 0.20; P = 0.40. Statistical significance was determined by a two-tail t test of ΔΔCt and difference from control is depicted by an asterisk. Oligonucleotide Primers from Applied Biosystems Inc., Foster City, CA were used: VEGF-A (Rn01511602_m1); VEGF-B (Rn01454585_g1); β-actin (Rn00667869_m1). The TaqMan^® ^MGB probe used at a concentration of 5 μM, comprised of a FAM™ reporter dye at the 5' end and a nonfluorescent quencher at the 3' end of the probe. The solution also contained the necessary primers at a concentration of 18 μM each.

Thirty-eight genes were up-regulated (using adjusted P = 0.025 and B = 2.5 as cutoff value; Table 1) after the rotenone treatment, and transcriptional activation of VEGF-B, but not of VEGF-A was evident. The results from the gene chip array experiment were verified with real time PCR (Figure 1), using β-actin as internal standard. Oligonucleotide primer sets that had been previously optimized for quantitative PCR. The PCR was perfomed on an ABI 7300 system (Applied Biosystems Inc., Foster City, CA).

**Table 1:** List of the highest up-regulated genes in the gene array after rotenone treatment

**gene_id**	**ID**	**adj. P value**	**B value**
			
**Atf5**	NM_172336	3.39E-05	9.733544
**RGD1561519_predicted**	NM_001109112	0.003306	6.712279
**Asns**	U07201	0.003466	6.388728
**LOC690315**	NM_001109577	0.004486	5.986556
**Slc7a5**	NM_017353	0.006186	5.572276
**Sdf2l1_predicted**	NM_001109433	0.007716	5.109109
**Lce1s_predicted**	XM_001066389	0.007716	5.029038
**Slc7a3**	NM_017217	0.0086	4.713449
**Klhl6_predicted**	NM_001105867	0.0086	4.684208
**Ns5atp9**	NM_201418	0.008757	4.59833
**Mrc1_predicted**	NM_001106123	0.009356	4.427766
**Lrrc33**	NM_001024995	0.009356	4.41691
**LOC290651**	NM_001013880	0.010522	4.201058
**Pycs_predicted**	NM_001108524	0.010522	4.140525
**Vegfb**	NM_053549	0.010522	4.090442
**Shmt2**	NM_001008322	0.010522	4.077214
**Ncf1**	NM_053734	0.010522	4.063953
**Aldh1l2_predicted**	XM_235005	0.01345	3.812624
**Mmp9**	NM_031055	0.015332	3.603003
**Dnajc3**	NM_022232	0.015332	3.583457
**Dpp7**	NM_031973	0.015332	3.516206
**Hmgcr**	NM_013134	0.015332	3.509113
**Gpnmb**	NM_133298	0.015786	3.39472
**Cndp2**	NM_001010920	0.015786	3.37061
**Pcp4**	M24852	0.015786	3.355451
**LOC680308**	NM_001109398	0.016276	3.29948
**Txnip**	NM_001008767	0.017441	3.210653
**Eprs**	NM_001024238	0.018104	3.150337
**Tm6sf1_predicted**	NM_001108490	0.019731	3.047957
**Calr**	NM_022399	0.020863	2.959711
**RGD1564553_predicted**	XM_577290	0.021343	2.854501
**Yars**	NM_001025696	0.02214	2.778994
**RGD1564228_predicted**	XM_001081442	0.02214	2.776624
**Igf1**	NM_178866	0.022922	2.701602
**Hexb**	NM_001011946	0.022922	2.681626
**Myo1f_predicted**	NM_001108076	0.023081	2.655012
**Cd180_predicted**	NM_001106405	0.024702	2.574536
**Cd68**	NM_001031638	0.025665	2.501227

List of the top 38 up-regulated genes in the gene array after rotenone treatment (genes with significance values of both the adjusted P ≤ 0.025 and the false discovery rate log-odds score B>2.5 were included in the Table; n = 3 separate experiments). The P value is based upon a t test of the average ratio of expression values for the treated and untreated samples and adjusted for multiple testing using the false discovery rate, as implemented in the Bioconductor limma library http://www.bioconductor.org. B is the false discovery rate expressed as a log odds score, so B = 3 means that there is 5% chance of being incorrect. VEGF-B is ranked 15^th ^highest, and the B = 4.09 correlates to >98% chance that treatment is different form control. MMP9 is ranked 19^th^, and the B value = 3.60 correlates to >97% chance that treatment is different form control.

**Figure 2:**
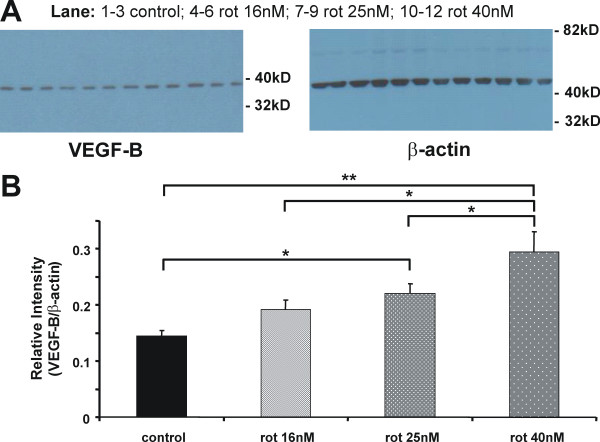
**Semi-quantitative western analysis of VEGF-B expression**. Midbrain cultures were exposed to 3 concentration of rotenone (16 nM, 25 nM and 40 nM) for 5 days. Protein was isolated and a western analysis was performed using a procedure previously published [32]. We used the expression of the house keeping β-actin protein (anti-β-actin antibody from Sigma-Aldrich, St. Louis, MO) as the internal standard for the relative quantification of VEGF-B protein levels (anti-VEGF-B antibody from Santa Cruz Biotechnology, Santa Cruz, CA). A concentration-dependent increase in VEGF-B protein level was evident. In (**A**) example western blots for VEGF-B (on the left) and β-actin (on the right) are shown; the samples were run in triplicate. This was repeated with 3 separate preparations. The summary graph in (**B**) depicts the mean relative VEGF-B level (± s.e.m.) that was determined by densitometry using Image-J (Wayne Rasband, Bethesda, MD). Statistical significant differences from control were determined by one-way ANOVA followed by a Fisher LSD post hoc test, and are depicted by asterisks (*P < 0.05, **P < 0.001). 40 nM rotenone induced a significantly greater increase than 16 nM and 25 nM rotenone (*P < 0.05). The statistical analyses were conducted using SPSS software, version 16.0 for Windows (SPSS, Chicago, IL).

To further validate these results a semi-quantitative western analysis was conducted. This experiment demonstrated an increase in protein expression of VEGF-B relative to the housekeeping β-actin protein after rotenone reaching significance at 25 nM rotenone. The highest rotenone concentration (40 nM) did lead to the highest expression of VEGF-B (Figure 2A and 2B).

With all techniques we saw a significant increase of VEGF-B after rotenone challenge, while there wass no significant change in VEGF-A mRNA level. This struck our interest in light of the recently published results from Poesen et al., 2008 [15], proving VEGF-B to be an inducible trophic factor in a model of neurodegeneration of motor neurons.

VEGF-B is a member of the VEGF-family of trophic factors [16,17]. VEGF-A is the best studied member due to its strong angiogenic activity and potential for cardiovascular and cancer research [18]. VEGF-A is up-regulated in the substantia nigra but not in the striatum of PD patients [19]. VEGF-B, on the other hand, stimulates proliferation of neuronal cultures *in vitro *[20], and has not been investigated in PD. VEGF-B has also recently been shown to be neuroprotective in motor neuron degeneration *in vivo *[15]. It had previously been shown to inhibit apoptosis and having only minimal angiogenic activity [21] while being critical to survival of the blood vessels [22]. This is important since efforts to use VEGF-A as a neurotrophic or a neuroprotective factor had been hampered by the strong angiogenic activity. Although neuroprotective effects of VEGF-A expressed by cells or viral vectors in models of PD were reported [23-25], they were over-shadowed by detrimental effects such as edema, ventriculomegaly [26] and disruption of the blood brain barrier [27]. These negative side effects were not seen when using VEGF-B *in vivo *[15]. Interestingly, the neuroprotective effect of VEGF-B *in vivo *was also restricted to pathological conditions. Mice lacking VEGF-B displayed normal motor behavior, but, when challenged in a model of neurodegeneration, they displayed a more severe form of motor degeneration [15]. Loss of VEGF-B also enlarges stroke [20]. These data suggest that VEGF-B plays a role in compensations for natural disease processes of the nervous system. It does so by binding to its only receptor VEGFR1 [18], a receptor with not yet delineated downstream signaling events. Further analysis of our gene array data showed the only up-regulated gene with known interaction with VEGF-B was matrix metallopeptidase 9 (MMP9). VEGFR1 signaling has been previously linked to the induction of MMP9 in lung endothelial cells [28] suggesting a potential role of MMP9 in the effects of VEGF-B that should be further investigated.

The fact that dopaminergic neurons make up less than 5% of the cells in our midbrain preparation argues against an up-regulation of VEGF-B only in dopaminergic neurons. We therefore hypothesize that the VEGF-B may be released by the astroglia in the preparation rather than the dopaminergic neurons themselves. Further evidence supporting this hypothesis comes from recent experiments where rotenone treatment *in vivo *did not cause transcriptional activation of VEGF-B in dopaminergic neurons analyzed after laser-capture microdissection [29]. In addition, under healthy conditions, motor neurons express VEGF-B to maintain neuroprotection in an autocrine manner, whereas astrocytes may express VEGF-B after injury to maintain survival in a paracrine manner [15]. A similar paracrine scenario is possible in the PD-like neurodegeneration induced in our model system. This hypothesis should be tested in the future. Taken together with our data showing an up-regulation of VEGF-B after rotenone challenge to rat midbrain cultures, these data lead to our hypothesis that VEGF-B may act as an endogenous trophic factor against the neurodegenerative insult in a model of PD.

**Figure 3:**
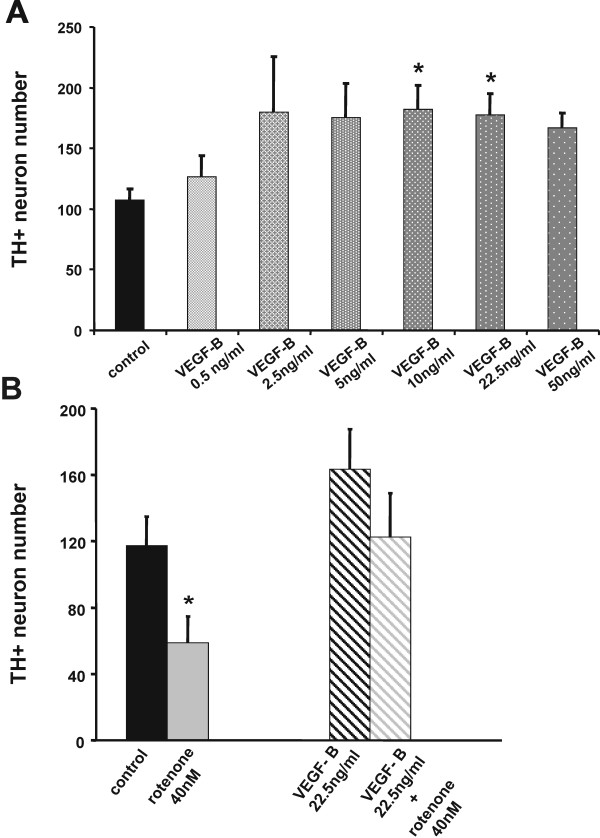
**(A) Neurotrophic effect of VEGF-B_167 _on cultured rat midbrain dopaminergic neurons**. Mean count (± s.e.m.) of TH-positive neurons after immunocytochemical staining is plotted. Fluorescent images were acquired digitally on an Olympus IX70 inverted microscope and camera using Olympus MagnaFire software. Morphometric analysis was carried out using Image-J (Wayne Rasband, Bethesda, MD). Antibodies were obtained from Chemicon, Temecula, CA. As compared to untreated control cultures (n = 22 culture dishes) a significant increase in the TH-positive neuron number per culture dish after addition of VEGF-B was apparent. The effect of VEGF-B on TH-positive cell number was concentration dependent. At 0.5 ng/ml (n = 8) no change was noticeable. At 2.5 ng/ml (n = 6) and 5 ng/ml (n = 10) a trend was evident, that reached a statistical significant level at 10 ng/ml (n = 12) and 22.5 ng/ml (n = 11). The mean effect remained the same at the highest tested concentration at 50 ng/ml (n = 4, P = 0.051). Culture dishes were from 5 separate preparations. Data are plotted as mean ± s.e.m. (*P < 0.05; one-way ANOVA followed by a Games-Howell post hoc test to account for small group size and heterogeneity of variance). The statistical significant differences from control are depicted by asterisks. **(B) Neuroprotective effect of VEGF-B in a severe rotenone rat midbrain *in vitro *PD model**. As compared to the untreated control cultures (n = 17; black bar) the TH-positive neuron number was reduced after addition of rotenone (40 nM; n = 11; gray bar on the left), this cell loss was rescued by adding 22.5 ng/ml VEGF-B_167 _(VEGF-B+40 nM rotenone; n = 9; gray patterned bar on the right) prior to rotenone (22.5 ng/ml VEGF-B only cultures; n = 9; black patterned bar). Data are plotted as mean ± s.e.m. (*P < 0.05; one-way ANOVA followed by a Fisher LSD post hoc test). The statistical significant difference from control is depicted by an asterisk. Culture dishes from 5 separate preparations were used.

We investigated the trophic activity further by adding exogenous VEGF-B_167 _(0.5-50 ng/ml; R&D-Systems Inc., Minneapolis, MN) to midbrain cultures on day 6 (VEGF-B_167 _is the more abundant of 2 splice isoforms, VEGF-B_186 _being the other). The cultures were fixed on day 11 and dopaminergic cells were analyzed by tyrosine hydroxylase (TH) immunocytochemical staining [11]. Neuronal numbers were determined by visual observation of randomly chosen fields at 400× magnification, viewed with fluorescent optics to determine immunostained dopaminergic neurons and with phase contrast to determine total cell number. We saw a mean treatment effect of 30% increase in TH-positive cell number per culture dish as compared to the untreated cells, with a trend apparent at 2.5 ng/ml, and statistical significance at 10 ng/ml. The mean effect remained the same at the highest tested concentration at 50 ng/ml (Figure 3A). An experiment using 5 ng/ml of VEGF-B_186 _showed also a significant effect (data not shown). This is likely due to improvement in the native *in vitro *survival of the dopaminergic neurons under artificial culture conditions. Over the course of time *in vitro*, the total number of neurons usually decreases. This is characteristic of primary neurons in culture and likely reflects normal developmental apoptosis and the absence of the full complement of neurotrophic factors. A less likely explanation could be stimulation of neurogenesis, but this has not been demonstrated in the substantia nigra as it has been for the subgranular zone of the hippocampal dentate gyrus [30]. In a second series of experiments we tested additional exogenous VEGF-B in the midbrain culture model using a severe challenge. Exposure to rotenone (40 nM) for 5 days in culture led to a significant reduction of TH-positive neurons per culture dish by 50% (P < 0.05). A neuroprotective effect against the rotenone challenge on TH-positive neurons was evident (Figure 3B) with pretreatment with VEGF-B_167 _(22.5 ng/ml) 1 hour prior to the toxin.

Neurotrophic factors are promising agents to provide disease modification for PD. This report demonstrates that VEGF-B is a new candidate trophic factor distinct from the GDNF-family of trophic factors, and is activated by neurodegenerative challenges to the midbrain. Strengthening this natural protective response by either adding exogenous VEGF-B or up-regulating the endogenous VEGF-B levels may have the potential to be a disease modifying therapy for PD. Based on the literature, the VEGF-B_186 _isoform is more diffusible than VEGF-B_167 _*in vivo *[15,31], and therefore may have a greater therapeutic potential. We conclude that the growth factor VEGF-B can improve neuronal survival in a culture model of PD.

## Abbreviations

VEGF-A: vascular endothelial growth factor A; VEGF-B: vascular endothelial growth factor B; VEGFR1: vascular endothelial growth factor receptor 1; GDNF: glial-derived neurotrophic factor; TH: tyrosine hydroxylase; PD: Parkinson's disease; MMP9: matrix metallopeptidase 9; IACUC: Institutional Animal Care and Use Committee.

## Competing interests

The authors declare that they have no competing interests.

## Authors' contributions

TF helped to design the study, analyze data and wrote the manuscript. SZ executed the experiments and helped with design and data analysis. SJS designed the study and contributed to writing the manuscript. All authors read and approved the final manuscript.

## References

[B1] Obeso JA Rodriguez-Oroz MC Rodriguez M Lanciego JL Artieda J Gonzalo N Olanow CW Pathophysiology of the basal ganglia in Parkinson's disease Trends Neurosci. 2000 23 10 Suppl S8 S19 10.1016/S1471-1931(00)00028-8 11052215

[B2] Stocchi F Olanow CW Neuroprotection in Parkinson's disease: clinical trials Ann Neurol. 2003 53 Suppl 3 S87 S97 discussion S97-9 10.1002/ana.10488 12666101

[B3] Fahn S Sulzer D Neurodegeneration and neuroprotection in Parkinson disease NeuroRx 2004 1 1 139 154 10.1602/neurorx.1.1.139 15717014 PMC534919

[B4] Savitt JM Dawson VL Dawson TD Diagnosis and treatment of Parkinson's disease: molecules to medicine J Clin Invest 2006 116 7 1744 1754 10.1172/JCI29178 16823471 PMC1483178

[B5] Peterson AL Nutt JG Treatment of Parkinson's disease with trophic factors Neurotherapeutics. 2008 5 2 270 280 10.1016/j.nurt.2008.02.003 18394569 PMC5084169

[B6] Grothe C Timmer M The physiological and pharmacological role of basic fibroblast growth factor in the dopaminergic nigrostriatal system Brain Res Rev 2007 54 1 80 91 10.1016/j.brainresrev.2006.12.001 17229467

[B7] Kirik D Georgievska B Björklund A Localized striatal delivery of GDNF as a treatment for Parkinson disease Nature Neurosci 2004 7 2 105 110 10.1038/nn1175 14747832

[B8] Ceregene Announces Clinical Data from Phase 2 Clinical Trial of CERE-120 for Parkinson's Disease http://www.medicalnewstoday.com/articles/130981.php

[B9] Bove J Prou D Perier C Przedborski S Toxin-Induced Models of Parkinson's Disease NeuroRx 2005 2 484 494 10.1602/neurorx.2.3.484 16389312 PMC1144492

[B10] Meredith GE Sonsalla PK Chesselet MF Animal models of Parkinson's disease progression Acta Neuropathol 2008 115 4 385 398 10.1007/s00401-008-0350-x 18273623 PMC2562277

[B11] Falk T Zhang S Sherman SJ Pigment epithelium derived factor (PEDF) is neurotrophic and neuroprotective in two *in vitro *models of Parkinson's disease Neurosci Lett 2009 458 49 52 10.1016/j.neulet.2009.04.018 19442875

[B12] McKay BS Goodman B Falk T Sherman SJ Retinal pigment epithelial cell transplantation could provide trophic support in Parkinson's disease: results from an in vitro model system Exp Neurol 2006 201 1 234 243 10.1016/j.expneurol.2006.04.016 16764861

[B13] Mount D Bioinformatics: sequence and genome analysis 2004 Sec. Cold Spring Harbor Press

[B14] Smyth GK Gentleman R, Carey V, Dudoit S, Irizarry R, Huber W Limma: linear models for microarray data Bioinformatics and Computational Biology Solutions using R and Bioconductor 2005 New York: Springer 397 420 full_text

[B15] Poesen K Lambrechts D Van Damme P Dhondt J Bender F Frank N Bogaert E Claes B Heylen L Verheyen A Raes K Tjwa M Eriksson U Shibuya M Nuydens R Bosch L Van Den Meert T D'Hooge R Sendtner M Robberecht W Carmeliet P Novel role for vascular endothelial growth factor (VEGF) receptor-1 and its ligand VEGF-B in motor neuron degeneration J Neurosci 2008 28 42 10451 10459 10.1523/JNEUROSCI.1092-08.2008 18923022 PMC6671326

[B16] Rosenstein JM Krum JM New roles for VEGF in nervous tissue--beyond blood vessels Exp Neurol 2004 187 246 253 10.1016/j.expneurol.2004.01.022 15144851

[B17] Roya H Bhardwaja S Ylä-Herttuala S Biology of vascular endothelial growth factors FEBS Lett 2006 580 2879 2887 10.1016/j.febslet.2006.03.087 16631753

[B18] Olsson AK Dimberg A Kreuger J Claesson-Welsh L VEGF receptor signalling - in control of vascular function Nat Rev Mol Cell Biol 2006 7 5 359 371 10.1038/nrm1911 16633338

[B19] Wada K Arai H Takanashi M Fukae J Oizumi H Yasuda T Mizuno Y Mochizuki H Expression levels of vascular endothelial growth factor and its receptors in Parkinson's disease Neuroreport 2006 17 7 705 709 10.1097/01.wnr.0000215769.71657.65 16641673

[B20] Sun Y Jin K Childs JT Xie L Mao XO Greenberg DA Increased severity of cerebral ischemic injury in vascular endothelial growth factor-B-deficient mice J Cereb Blood Flow Metab 2004 24 1146 1152 10.1097/01.WCB.0000134477.38980.38 15529014

[B21] Li Y Zhang F Nagai N Tang Z Zhang S Scotney P Lennartsson J Zhu C Qu Y Fang C Hua J Matsuo O Fong GH Ding H Cao Y Becker KG Nash A Heldin CH Li X VEGF-B inhibits apoptosis via VEGFR-1-mediated suppression of the expression of BH3-only protein genes in mice and rats J Clin Invest 2008 118 913 923 10.1172/JCI33637C1 18259607 PMC2230661

[B22] Zhang F Tang Z Hou X Lennartsson J Li Y Koch AW Scotney P Lee C Arjunan P Dong L Kumar A Rissanen TT Wang B Nagai N Fons P Fariss R Zhang Y Wawrousek E Tansey G Raber J Fong GH Ding H Greenberg DA Becker KG Herbert JM Nash A Yla-Herttuala S Cao Y Watts RJ Li X VEGF-B is dispensable for blood vessel growth but critical for their survival, and VEGF-B targeting inhibits pathological angiogenesis Proc Natl Acad Sci USA 2009 106 15 6152 6157 10.1073/pnas.0813061106 19369214 PMC2669337

[B23] Pitzer MR Sortwell CE Daley BF McGuire SO Marchionini D Fleming M Collier TJ Angiogenic and neurotrophic effects of vascular endothelial growth factor (VEGF165): studies of grafted and cultured embryonic ventral mesencephalic cells Exp Neurol 2003 182 435 445 10.1016/S0014-4886(03)00100-6 12895454

[B24] Yasuhara T Shingo T Kobayashi K Takeuchi A Yano A Muraoka K Matsui T Miyoshi Y Hamada H Date I Neuroprotective effects of vascular endothelial growth factor (VEGF) upon dopaminergic neurons in a rat model of Parkinson's disease Eur J Neurosci. 2004 19 6 1494 1504 10.1111/j.1460-9568.2004.03254.x 15066146

[B25] Tian YY Tan CJ Wang JN Feng Y Chen XW Wang L Qiao X Sun SG Favorable effects of VEGF gene transfer on a rat model of Parkinson disease using adeno-associated viral vectors Neurosci Lett 2007 421 3 239 244 10.1016/j.neulet.2007.05.033 17574749

[B26] Harrigan MR Ennis SR Sullivan SE Keep RF Effects of intraventricular infusion of vascular endothelial growth factor on cerebral blood flow, edema, and infarct volume Acta Neurochir (Wien) 2003 145 49 53 10.1007/s00701-002-1035-1 12545262

[B27] Rite I Machado A Cano J Venero JL Blood-brain barrier disruption induces in vivo degeneration of nigral dopaminergic neurons J Neurochem 2007 101 6 1567 1582 10.1111/j.1471-4159.2007.04567.x 17437543

[B28] Ferrara N Vascular endothelial growth factor: basic science and clinical progress Endocr Rev 2004 25 4 581 611 10.1210/er.2003-0027 15294883

[B29] Meurers BH Zhu C Fernagut PO Richter F Hsia YC Fleming SM Oh M Elashoff D Dicarlo CD Seaman RL Chesselet MF Low dose rotenone treatment causes selective transcriptional activation of cell death related pathways in dopaminergic neurons in vivo Neurobiol Dis. 2009 33 2 182 192 10.1016/j.nbd.2008.10.001 19013527 PMC3731054

[B30] Sun Y Kunlin J Childs JT Xie L Mao XO Greenberg DA Vascular endothelial growth factor-B (VEGFB) stimulates neurogenesis: Evidence from knockout mice and growth factor administration Develop Biol 2006 289 329 335 10.1016/j.ydbio.2005.10.016 16337622

[B31] Olofsson B Pajusola K von Euler G Chilov D Alitalo K Eriksson U Genomic organization of the mouse and human genes for vascular endothelial growth factor B (VEGF-B) and characterization of a second splice isoform J Biol Chem 1996 271 19310 19317 10.1074/jbc.271.32.19310 8702615

[B32] Falk T Xie JY Zhang SL Kennedy J Bennett J Yool AJ Sherman SJ Over-expression of the potassium channel Kir2.3 using the dopamine-1 receptor promoter selectively inhibits striatal neurons Neuroscience. 2008 155 1 114 127 10.1016/j.neuroscience.2008.04.075 18571331

